# [^18^F]FDG PET/MRI enables early chemotherapy response prediction in pancreatic ductal adenocarcinoma

**DOI:** 10.1186/s13550-021-00808-4

**Published:** 2021-07-28

**Authors:** Felix N. Harder, Friederike Jungmann, Georgios A. Kaissis, Fabian K. Lohöfer, Sebastian Ziegelmayer, Daniel Havel, Michael Quante, Maximillian Reichert, Roland M. Schmid, Ihsan Ekin Demir, Helmut Friess, Moritz Wildgruber, Jens Siveke, Alexander Muckenhuber, Katja Steiger, Wilko Weichert, Isabel Rauscher, Matthias Eiber, Marcus R. Makowski, Rickmer F. Braren

**Affiliations:** 1 Institute of Diagnostic and Interventional Radiology, School of Medicine, Technical University of Munich, Munich, Germany; 2 Department of Computing, Faculty of Engineering, Imperial College of Science, Technology and Medicine, London, SW7 2AZ UK; 3 Internal Medicine II, Faculty of Medicine, Freiburg University Hospital, Freiburg, Germany; 4 Klinik und Poliklinik für Innere Medizin II, Klinikum rechts der Isar, Technical University of Munich, Munich, Germany; 5 German Cancer Consortium (DKTK), Partner Site Munich, Munich, Germany; 6 Department of Surgery, School of Medicine, Klinikum rechts der Isar, Technical University of Munich, Munich, Germany; 7 Klinik und Poliklinik für Radiologie, Klinikum der Universität München, Munich, Germany; 8 Institute for Developmental Cancer Therapeutics, West German Cancer Center, University Hospital Essen, Essen, Germany; 9 Institute of Pathology, Technical University of Munich, Munich, Germany; 10 Department of Nuclear Medicine, Technical University Munich, Klinikum rechts der Isar, Munich, Germany

**Keywords:** PDAC, PET/MRI, Chemotherapy, Response prediction

## Abstract

**Purpose:**

In this prospective exploratory study, we evaluated the feasibility of [^18^F]fluorodeoxyglucose ([^18^F]FDG) PET/MRI-based chemotherapy response prediction in pancreatic ductal adenocarcinoma at two weeks upon therapy onset.

**Material and methods:**

In a mixed cohort, seventeen patients treated with chemotherapy in neoadjuvant or palliative intent were enrolled. All patients were imaged by [^18^F]FDG PET/MRI before and two weeks after onset of chemotherapy. Response per RECIST1.1 was then assessed at 3 months [^18^F]FDG PET/MRI-derived parameters (MTV_50%_, TLG_50%_, MTV_2.5_, TLG_2.5_, SUV_max_, SUV_peak_, ADC_max_, ADC_mean_ and ADC_min_) were assessed, using multiple *t*-test, Man–Whitney-*U* test and Fisher’s exact test for binary features.

**Results:**

At 72 ± 43 days, twelve patients were classified as responders and five patients as non-responders. An increase in ∆MTV_50%_ and ∆ADC (≥ 20% and 15%, respectively) and a decrease in ∆TLG_50%_ (≤ 20%) at 2 weeks after chemotherapy onset enabled prediction of responders and non-responders, respectively. Parameter combinations (∆TLG_50%_ and ∆ADC_max_ or ∆MTV_50%_ and ∆ADC_max_) further improved discrimination.

**Conclusion:**

Multiparametric [^18^F]FDG PET/MRI-derived parameters, in particular indicators of a change in tumor glycolysis and cellularity, may enable very early chemotherapy response prediction. Further prospective studies in larger patient cohorts are recommended to their clinical impact.

**Supplementary Information:**

The online version contains supplementary material available at 10.1186/s13550-021-00808-4.

## Introduction

Despite extensive research on therapeutic approaches, pancreatic ductal adenocarcinoma (PDAC) remains a tumor entity with high mortality rates, reflected in a 5-year survival rate of only 9% [1]. Furthermore, incidence of PDAC is rising in developed countries and thus PDAC is predicted to be the second leading cause of cancer-related death in the US by 2030 [2]. Surgical options are limited and disease relapse is frequent [3–6]. Systemic chemotherapy also plays a pivotal role in the therapy of advanced PDAC despite considerable low response rates and primary resistance in approximately 25% of patients to first line therapies [4, 7–9].

Moreover, second-line therapeutic options are limited with poor response rates, ranging between 2 and 10 months [10–13]. However, differential intraindividual response to standard therapy is observed [14–17]. Thus, early response assessment to detect non-responders and timely terminate ineffective chemotherapy especially in light of overall short survival times is warranted [4]. Reliable detection of non-responders would provide a rationale not only for a switch to commonly applied alternative regimen but also for the evaluation of novel therapeutics. They are often applied in an advanced disease stage in an overall compromised patient collective, thereby increasing toxicities, limiting efficacy due to acquired therapy resistance properties and introducing bias when comparing efficacy to first line therapies of untreated tumors [18, 19]. Particularly in locally advanced pancreatic cancer (LAPC) early chemotherapeutic response assessment is of utmost importance to enable identification of responders, i.e. resectability [20].

In clinical routine, for practicality reasons therapeutic response is monitored by computed tomography (CT) despite the known discrepancy and sometimes major delay observed between biological response and morphological response [21–25].

Functional imaging techniques like diffusion weighted magnetic resonance imaging (DWI) and metabolic imaging by [^18^F]fluorodeoxyglucose ([^18^F]FDG) PET/MRI are recently reported to potentially allow for a more accurate response prediction and evaluation in PDAC [26, 27].

Combining functional with metabolic imaging parameters, PET/MRI allows for simultaneous assessment of these potential biomarkers and the identification of potentially additive effects on the accuracy of therapeutic response prediction as previously reported in gastroesophageal junction cancer [28].

In this study, we prospectively evaluated the potential of multiparametric [^18^F]FDG PET/MRI in early chemotherapy response prediction in pancreatic cancer.

## Material and methods

### Study design and patients

This was a single center, single-arm, open-label, prospective exploratory study. Patients with biopsy proven pancreatic ductal adenocarcinoma who were scheduled to undergo chemotherapy either prior to intended surgical excision or for systemic treatment in palliative intention were offered participation in this study. Exclusion criteria were inability to tolerate a PET/MRI scan, other types of pancreatic tumor than PDAC, other concurrent malignant conditions in the last 10 years and prior chemotherapy. All patients underwent two PET/MRI examinations, one before the 1st and one before the 2nd (FOLFIRINOX) or 3rd (gemcitabine-based) chemotherapy cycle, respectively. The STrengthening the Reporting of OBservational studies in Epidemiology (STROBE) flowchart is included in the supplementary material (A. 1) (Additional file 1: Table A.1).

The study was conducted in accordance with the Declaration of Helsinki. Approval by the local ethics committee (Protocol Nr. 181 17S, Ethikkommission der Fakultät für Medizin der Technischen Universität München) was given and written informed consent was obtained from every patient.

### Clinical data

The following clinical data were obtained for all patients using the hospital’s information system: sex, age at diagnosis, initial tumor markers CEA and CA 19-9, tumor site (pancreatic head, body or tail), tumor grading, type of chemotherapy (neoadjuvant, palliative) and chemotherapeutic regime (FOLFIRINOX, gemcitabine-based). Clinical evaluation of the tumor size (T), lymph node status (N) and metastasis (M) was performed on baseline CT before starting the treatment. Response to chemotherapy was clinically evaluated at the first follow-up CT scan applying RECIST1.1 criteria [29]. According to the CT-findings patients were overall classified as responders (stable or regressive disease) or non-responders (progressive disease).

### Imaging protocol

All patients were instructed to fast for at least 6 h before the study. Serum glucose levels were controlled before [^18^F]FDG injection. In all patients, glucose levels were below 8.32 mmol/l.

Prior to image acquisition all patients received 20 mg furosemide for renal protection and 250 ml water for upper bowl distention. Simultaneous [^18^F]FDG PET/MRI was performed using an integrated whole-body 3T PET/MRI system (Siemens Biograph mMR, Siemens Healthcare, Erlangen, Germany). MRI examination of the pancreas was performed simultaneously within a 20-min list-mode PET acquisition of the upper abdomen. Initially, a T1-VIBE Dixon sequence was used for attenuation correction. Further MRI sequences included an axial and coronal T2 haste sequence, axial fat saturated (FS) T2 haste sequence, axial DWI (*b*-values 50, 300 and 600 s/mm^2^), axial dynamic T1 VIBE Dixon sequence (arterial, venous and late venous phase) in breath-hold before and after dynamic administration of contrast agent. Finally, a late post contrast axial T1 VIBE Dixon FS was acquired. Detailed sequence parameters are displayed in the supplement (Additional file 1: Table A.2). PET data were reconstructed using a vendor-provided iterative reconstruction algorithm (3 iterations, 21 subsets, image matrix 172 × 172, zoom 1, gauss filter, full width at half maximum 4.0 mm, relative scatter correction).

### Image analysis

Image analysis was performed by one radiologist with 3 years of experience (FH) under supervision of a board certified expert abdominal radiologist with 10 years of experience as well as a board certified expert nuclear medicine physician with 10 years of experience.

All images were analyzed using OsiriX (OsiriX DICOM viewer, 11.0 OsiriX Foundation; Geneva, Switzerland). The tumor was identified by reviewing the axial T2w, DWI and ADC together with the PET-images. The maximum as well as the peak standardized uptake values (SUV_max_ and SUV_peak_; in g/ml) were obtained for all tumors. SUV was normalized by total body weight. The metabolic tumor volume (MTV; in cm^3^) was assessed using the OsiriX-integrated 3D ROI application.

MTV_50%_ defines the tumor volume at a threshold of 50% of SUV_max_. MTV_2.5_ represents the tumor volume above a SUV threshold of 2.5 [30–32]. Total lesion glycolysis (TLG) values were automatically generated based on MTV_50%_ and MTV_2.5_ respectively (TLG_50%,_ TLG_2.5_).

Using the peak value option, a 10 mm ROI was automatically placed in the tumor area with the highest SUV value. This ROI was copied and pasted to the ADC map in the same image slice. Within this ROI ADC_min_, ADC_max_ and ADC_mean_ (10^−3^ mm^2^/s) were assessed.

In all patients, the maximum tumor diameter was measured in the axial T2-weighted sequence in both, the first and second PET/MRI scan and the percentage of change was calculated.

### Statistical modelling

All statistical analyses were performed in Python 3.7.6 with a two-sided level of significance of *p* < 0.05. Data were normalized to unity interval for better comparison and single missing continuous values were imputed using the median value. The two-sided t-test was used to analyze the change in PET/MRI features as well as ADC values between the first and second PET/MRI examination among responders and non-responders. Multiple testing correction was utilized in the form of Bonferroni correction. Furthermore, ROC analysis was applied to determine specific cut-off values to distinguish between responders and non-responders.

In a second step, feature combination was assessed, in order to better distinguish between responders and non-responders. Again, ROC analysis was performed for the obtained values.

To assess for potential confounders with regard to chemotherapeutic response, clinical data of all patients as well as the features of the first and second PET/MRI were analyzed using the Mann–Whitney *U* test. Binary data were analyzed using Fisher’s exact test.

## Results

### Patient characteristics

Between November 2018 and March 2020, fifty-four patients were examined for eligibility. Thirty-seven patients were excluded for following reasons: no available second PET/MRI (*n* = 16), missing follow-up CT (*n* = 12) and other pancreatic tumor than PDAC (*n* = 9). In total seventeen patients of UICC stages I-IV (Stage I *n* = 1; Stage II *n* = 1; stage III *n* = 5; stage IV *n* = 10). Detailed patient characteristics are displayed in Table 1. Last follow-up was 30^th^ of April 2020. Time intervals for chemotherapy and image acquisition are displayed in Table 2. Follow-up CT revealed progressive disease in five patients (29%), stable disease in four patients (24%) and regressive disease in eight patients (47%). Response to chemotherapy did not differ significantly between patients receiving chemotherapy in a neo-adjuvant (*n* = 6) versus palliative (*n* = 11) intent (*p* = 0.34). All patients with a borderline resectable tumor status were treated in neo-adjuvant intent. In the neoadjuvant cohort, chemotherapy led to a resectable tumor stage in 5 out of 6 patients and surgery was performed after the first follow up.

**Table 1:** displaying patient characteristics

	Variable	Responder (*n* = 12)	Non-Responder (*n* = 5)
Sex	Male	9 (75%)	1 (20%)
	Female	3 (25%)	4 (80%)
Age (years)	Mean ± SD	62 ± 5	70 ± 5
Tumour size	cT1	0 (0%)	0 (0%)
	cT2	3 (25%)	2 (40%)
	cT3	1 (8%)	0 (0%)
	cT4	8 (67%)	3 (60%)
Nodal status	cN0	5 (42%)	1 (20%)
	cN1	3 (25%)	2 (40%)
	cN2	4 (33%)	2 (40%)
Metastasis	cM0	6 (50%)	1 (20%)
	cM1	6 (50%)	4 (80%)
CA19-9 (U/ml)	Median	621	5044
	IQR	665	1241
CEA (ng/ml)	Median	3.3	38.0
	IQR	2.7	25.5
First line chemotherapy	FOLFIRINOX	7 (58%)	4 (80%)
	gemcitabine based	5 (42%)	1 (20%)

Statistical analyses were performed with a two-sided level of significance of* p* < 0.05

**Table 2:** displays imaging time intervals

Events	Mean time interval (days)
1st PET/MRI until start chemotherapy	3 ± 2
1st PET/MRI until 2nd PET/MRI	17 ± 3
Chemotherapy onset until 2nd PET/MRI	14 ± 3
1st PET/MRI until follow-up CT	72 ± 43

### Response assessment

Images were obtained after intravenous injection of a bodyweight adapted dose of [^18^F] fluorodeoxyglucose (FDG) (4.6 MBq/kg, mean 331.29 ± 51.69 MBq). PET/MRI scans were performed 70 ± 9 min (range 54–88 min) after tracer injection. Overall, three parameters, namely ∆MTV_50%,_ ∆ADC_mean_ and ∆TLG_50%_, were found to significantly and independently predict response to chemotherapy.

According to Bonferroni correction the level of significance was set to *α* = 0.0056. As shown in Table 3, patients who responded to chemotherapy showed a mean of 28 ± 1.8% reduction of MTV_50%_ compared to an increase of 126 ± 98% in non-responders (*p* < 0.0001). Furthermore, at ∆TLG_50%_ responders exhibited a decrease of 39 ± 14% versus an increase of 63 ± 112% in the non-responder cohort (*p* = 0.0054).

**Table 3:** Displays the change in the assessed imaging features between the first and second PET/MRI examination, for both chemotherapy responders and non-responders. Absolute values from the baseline as well as the follow-up PET/MRI can be found in the supplementary material (A. 2)

	Responder (Mean ± SD)	Non-responder (Mean ± SD	*p*-value
∆MTV_50%_	−0.28 ± 0.18	+ 1.26 ± 0.98	** < 0.0001**
∆ADC_mean_	+ 0.27 ± 0.10	+ 0.07 ± 0.09	**0.0011**
∆TLG_50%_	−0.39 ± 0.14	+ 0.63 ± 1.12	**0.0054**
∆SUV_max_	−0.24 ± 0.17	−0.33 ± 0.18	0.3372
∆SUV_peak_	−0.15 ± 0.13	−1.23 ± 0.25	0.8296
∆MTV_2,5_	−0.53 ± 0.24	+ 0.05 ± 0.98	0.0696
∆TLG_2,5_	−0.53 ± 0.26	−0.02 ± 1.1	0.1419
∆ADC_min_	+ 0.33 ± 0.26	−0.07 ± 0.21	0.0080
∆ADC_max_	0.24 ± 0.16	0.26 ± 0.14	0.7955

**Fig. 1:**
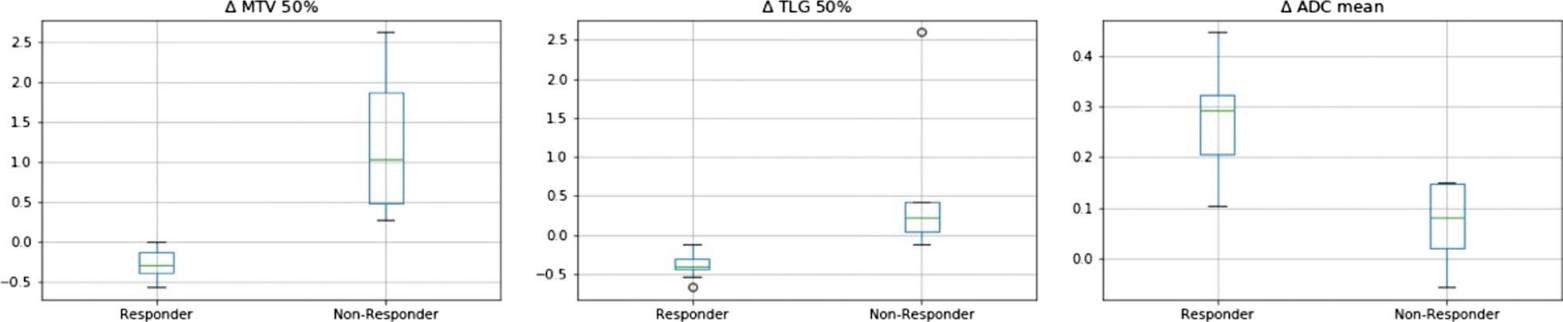
displays Box-plots for ∆MTV_50%_, ∆TLG_50%_ and ∆ADC_mean_ between chemotherapy responders and non-responders

Additionally, a significantly larger increase of ∆ADC_mean_ of 27 ± 10% was observed in responders compared to 7 ± 9% in non-responders (*p* = 0.0011). The boxplots for the significant features are shown in Fig. 1.

Mean tumor size at the initial PET/MRI was 3.1 ± 1.2 cm and 2.8 ± 0.94 cm at the second PET/MRI. A larger decrease in tumor size was detectable in responders (11 ± 6%) compared to non-responders (4 ± 3%), yet not reaching statistical significance (*p* = 0.452).

Based upon the results shown above, ROC analysis provided specific cut-off values to distinguish between responders and non-responders. The best cut-off values regarding the PET/MRI and ADC features are shown in Table 4.

**Table 4:** ∆MTV_50%_, ∆ADC_mean_ and ∆TLG_50%_ enable high sensitivity and specificity in separating between chemotherapy responders and non-responders

	Cut-off value	ROC-AUC	Sensitivity	Specificity
∆MTV_50%_	+ 0.20	1.00	1.00	1.00
∆ADC_mean_	+ 0.15	0.82	0.83	0.8
∆TLG_50%_	− 0.20	0.96	0.92	1.00

**Fig. 2:**
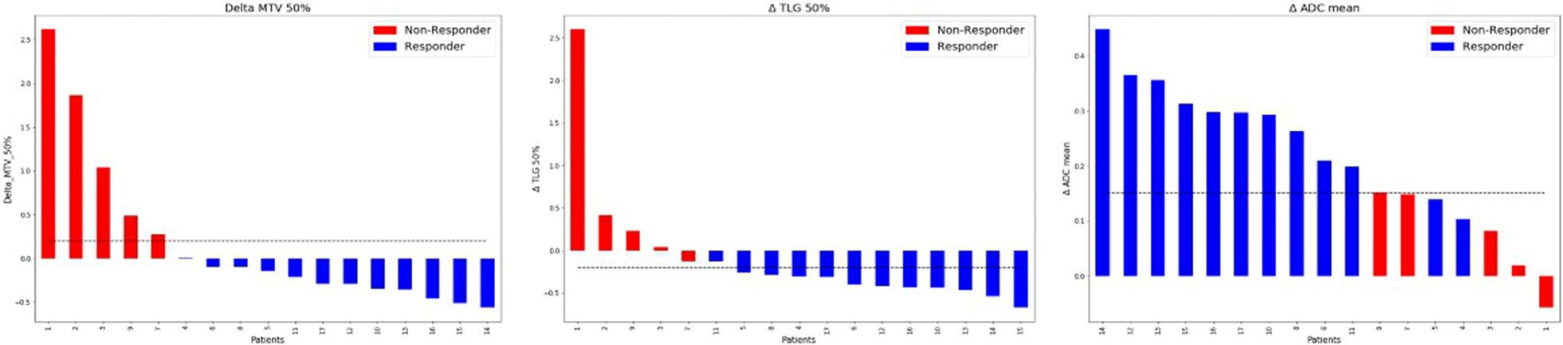
Waterfall plots demonstrating high sensitivity and specificity in differentiating between chemotherapy responders and non-responders for ∆MTV_50%_, ∆TLG_50%_, and ∆ADC_mean_

With a cut-off value of ∆MTV_50%_ =  + 20%, responders were perfectly distinguished from non-responders (ROC-AUC = 1.00, sensitivity = 1.00, specificity = 1.00). A cut-off value of ∆ADC_mean_ =  + 15% achieved a ROC-AUC of 0.82 with sensitivity = 0.83 and specificity = 0.80. For ∆TLG_50%_, a decrease of 20% yielded a ROC-AUC of 0.96 for distinguishing responder and non-responder with a sensitivity = 0.92 and specificity = 1.00. The waterfall plots of these three features are shown in Fig. 2.

The t-test with a level of significance of *α* = 0.0014 according to the Bonferroni correction yielded significant feature combinations for potential differentiation between responders and non-responders to chemotherapy. ROC analysis was performed like described above. The corresponding results are displayed in Table 5.

**Table 5:** Combination of PET/MRI features as well as the ADC allows differentiation between chemotherapy responders and non-responders with high sensitivity and specificity

	Cut-off value	ROC-AUC	Sensitivity	Specificity
∆TLG 50% *∆ADC max	0	0.86	0.92	0.8
∆SUVmax *∆MTV50%	0	1.00	1.00	1.00
∆SUVmax *∆TLG 50%	0	0.86	0.92	0.80
∆MTV50% *∆ADC max	0.10	1.00	1.00	1.00

A perfect differentiation between patients who responded to therapy and those who did not benefit could be achieved using the combination of ∆MTV_50%_ with either ∆ADC_max_ or ∆SUV_max_ (both ROC-AUC = 1.00, sensitivity = 1.00, specificity = 1.00). Here, the best cut-off value was an increase of 10% for the combination of ∆MTV_50%_ and ∆ADC_max_.

**Fig. 3:**
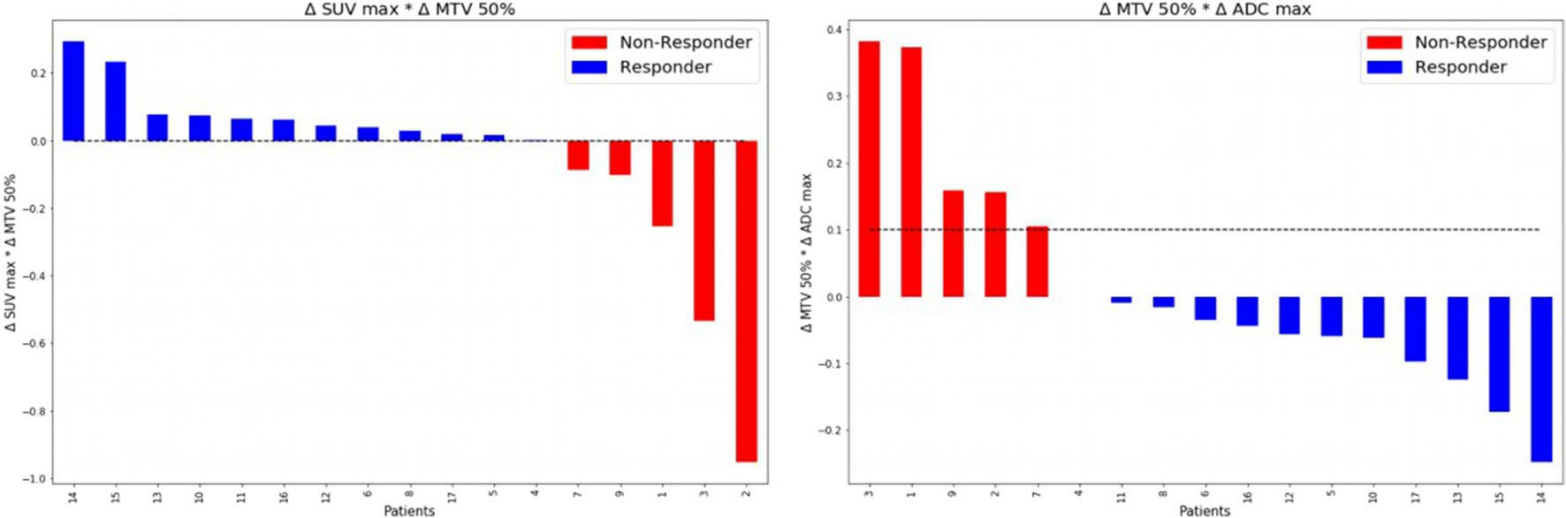
Combining ∆SUV_max_ and ∆MTV_50%_ as well as and ∆MTV_50%_ and ∆ADC_max_ enables perfect differentiation between chemotherapy responders versus non-responders

**Fig. 4:**
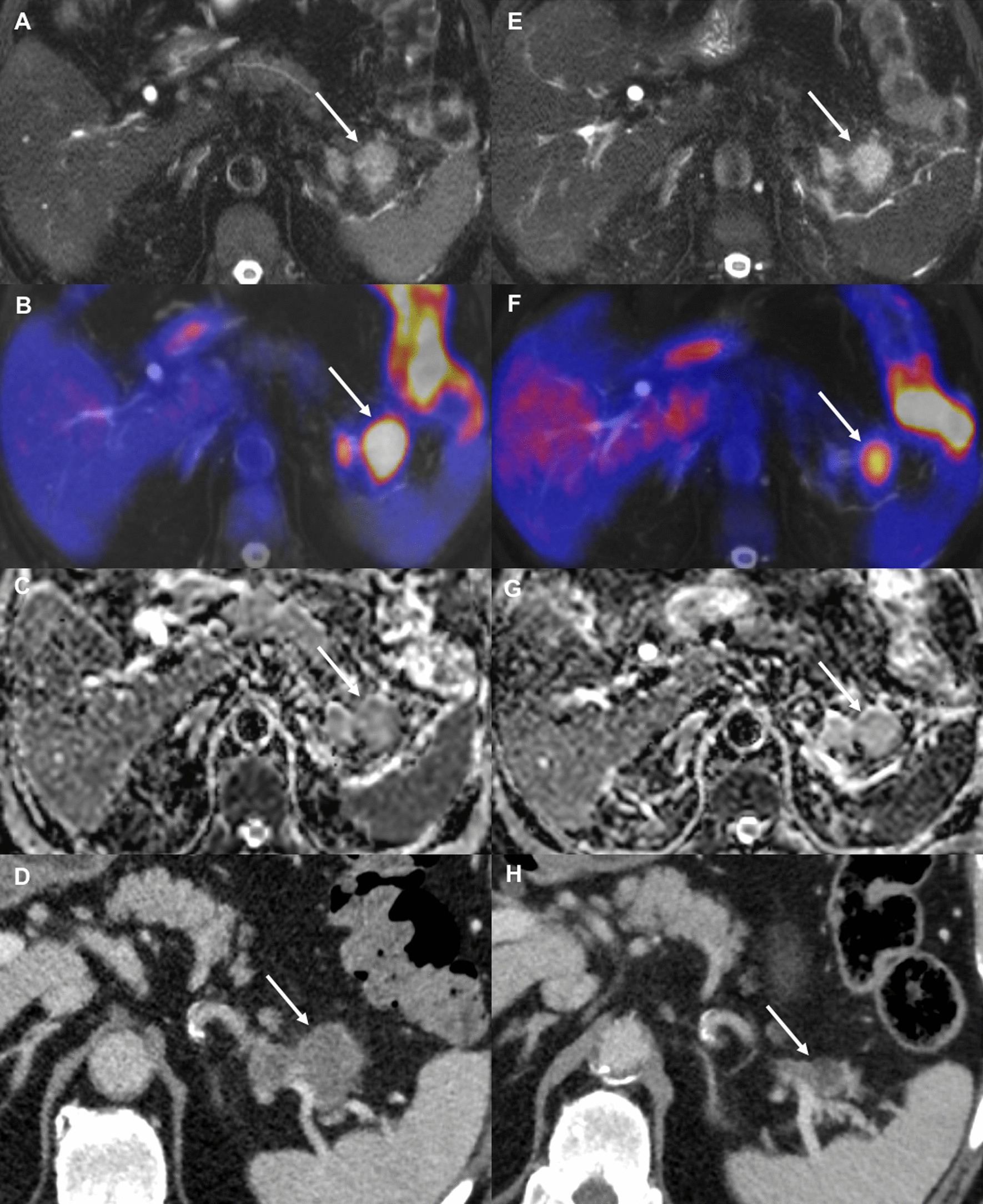
Images from a 69y-old male patient with a PDAC in the pancreatic tale. Images on the left (**a**–**d**) were obtained prior to the onset of chemotherapy, images on the right (**e**–**h**) were obtained 14 days later, after two cycles of neoadjuvant chemotherapy with nab-Paclitaxel/gemcitabine. Axial T2 FS (**a**, **e**) show no change in tumor size. However, PET showed a significant decrease in MTV_50%_ (56.4%) and TLG_50%_ (53.9%) (**b**, **f**). Furthermore, a significant increase in ADC_mean_ (44.8%) was seen (**c**, **g**). The bottom row displays the baseline CT (**d**) as well as the follow-up CT (**h**) after 5 cycles of nab-Paclitaxel/gemcitabine in the same patient. The follow-up CT was obtained after 81 days. Based on RECIST1.1 the patient was stratified as a responder. Left-sided pancreatic resection with splenectomy was performed after the follow-up CT

**Fig. 5:**
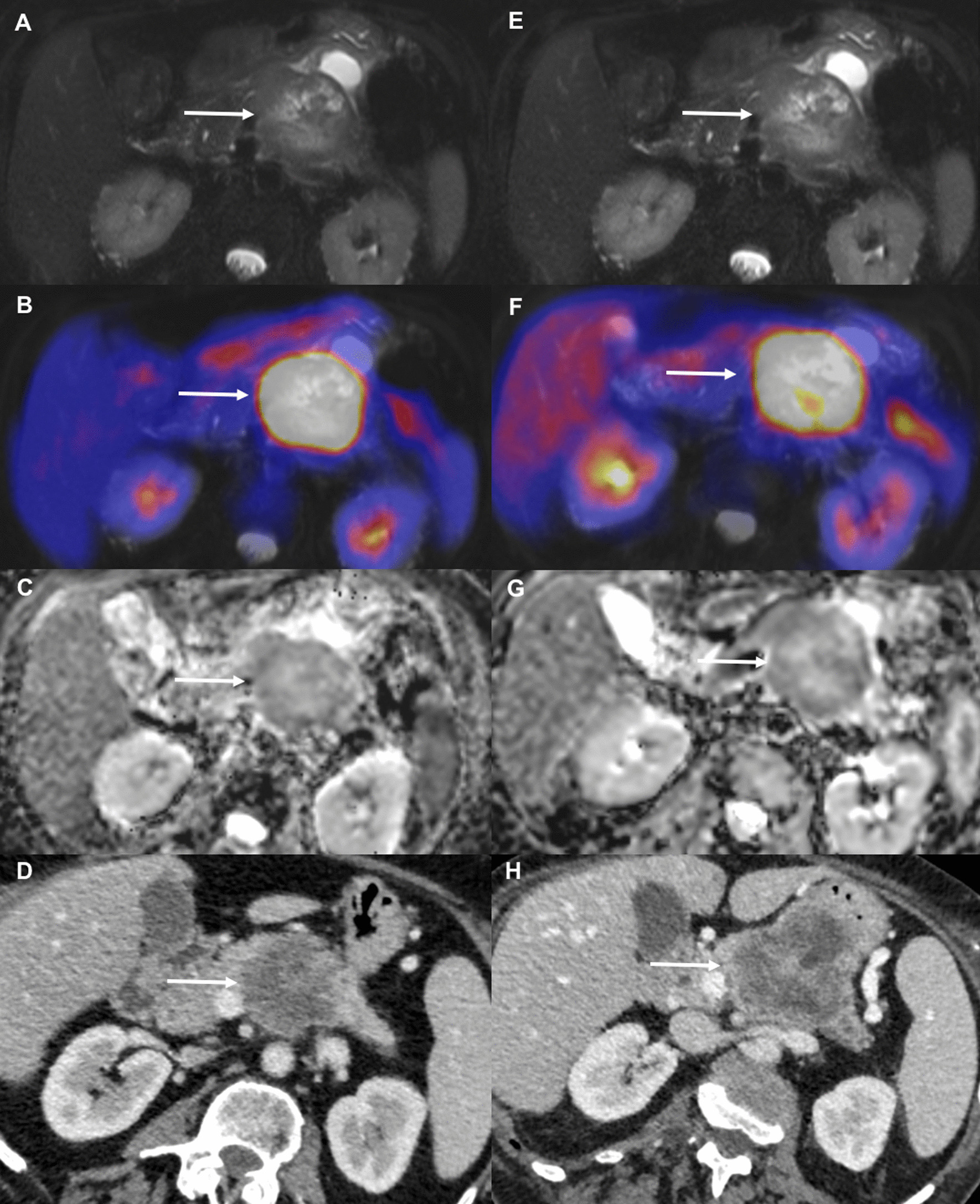
Images from a 66y-old female patient with a PDAC in the pancreatic corpus. Images on the left (**a**–**d**) were obtained prior to chemotherapy, images on the right (**e**–**h**) were obtained 14 days later, after one cycle of palliative chemotherapy with FOLFIRINOX. Axial T2 FS images (**a**, **e**) display no significant change in tumor size. However, PET showed a significant increase in MTV_50%_ (33.8%) and TLG_50%_ (19.9%) (**b**, **f**). Additionally, only a slight increase in ADC_mean_ (7%) was seen (**c**, **g**). The follow-up CT (**h**) after 78 days reveals progressive disease despite 6 cycles of FOLFIRINOX compared to the baseline CT (**d**)

Regarding the combination of ∆MTV_50%_ and ∆SUV_max_, the best cut-off value was zero, indicating the increase or decrease of this feature combination to be predictive for chemotherapy response. The waterfall plots for these feature combinations are displayed in Fig. 3. Figures 4 and 5 display changes in PET/MRI as well as the baseline CT and the follow-up CT in a responder and non-responder, respectively.

Possible confounders were assessed using the Man-Whitney U test for continuous and Fisher’s exact test for binary features. This revealed a significant difference between responders and non-responders in age (*p* = 0.0227) and the absolute value of MTV_50%_ of the second PET/MRI (*p* = 0.0177). Details of the analysis are provided in Additional file 1: Tables A. 3 and A. 4 of the supplement.

## Discussion

In this prospective exploratory study, we assessed the predictive performance of multiparametric [^18^F]FDG PET/MRI to assess response to chemotherapy in PDAC. Our results indicate that early changes in [[^18^F]FDG PET/MRI-derived biomarkers MTV_50%_, TLG_50%_ and ADC_mean_ enable differentiation between responders and non-responders.

In PDAC, common chemotherapy regimen with dose limiting adverse effects and generally low efficacy warrant early therapy response assessment to limit unnecessary impairment of quality of life. The same holds true for efficacy testing of new drugs. These are usually introduced after prolonged application and subsequent failure of standard drugs in an advanced disease stage. In such PDAC patient collectives, the overall performance status is often reduced and prolonged drug exposure has led to drug resistance and molecular alterations that may lead to negative trial results [33].

Few data on sequential [^18^F]FDG PET/MRI for prediction of response in PDAC have been reported in the literature so far. In our study, [^18^F]FDG PET/MRI was performed before and two weeks after therapy onset. In a prior similar study Wang et al. evaluated [^18^F]FDG PET/MRI before and 4 weeks after treatment initiation in advanced PDAC [23]. In accordance with our findings, the authors came to the conclusion that changes in [[^18^F]FDG PET/MRI -derived parameters MTV, TLG and ADC enabled early discrimination between responders and non-responders based on CT evaluation after 8–12 weeks, further underlining the potential role of early time point PET/MRI after treatment initiation. However, our data indicated the potential to predict therapy response after limited exposure (e.g. a single infusion of FOLFIRINOX). Due to the recent advancement in neoadjuvant therapy regimen, patients with LAPC would in particular profit from a more detailed therapy monitoring since in this patient collective neoadjuvant induction therapy aims at conversion to a resectable tumor stage [34–36].

For example, a conversion rate of 30.6% for LAPC patients treated with nab-paclitaxel/gemcitabine and 44% for patients treated with nab-paclitaxel/gemcitabine and sequential FOLFIRINOX was reported in the recently published NEOLAP study [20]. Therapy assessment using standard CT imaging is difficult in these patients since desmoplastic fibrosis often cannot be distinguished from viable tumor tissue [20, 37, 38].

Thus, careful and early reevaluation of therapy response especially in the neoadjuvant setting is of great interest to avoid prolonged and inefficient chemotherapy resulting in higher toxicity as well as delayed and less successful resection [27]. In our cohort six patients received neoadjuvant intended chemotherapy, five of which were resected after the first follow-up CT. According to RECIST 1.1, tumor size did not change significantly in any of those patients between both PET/MRI examinations while metabolism and cellularity parameters already indicated response to therapy in all patients.

In our study, we used fixed relative and absolute SUV thresholds of 50% and 2.5, as previously proposed [30–32]. High sensitivity and specificity were found for both volumetric parameters MTV_50%_ and TLG_50%_. Particularly MTV_50%_ enabled perfect discrimination between responders and non-responders. It is worth noting that a 50% SUV_max_ threshold bears the risk of overestimating the tumor volume of lesions with a low SUV_max_. Yet only one patient in our cohort had an SUV_max_ below 4 g/ml. In contrast to the recent publication by Wang et al., changes in MTV_2.5_ and TLG_2.5_ were not significantly associated with chemotherapy response in our study [23]. This might be due to the fact that absolute thresholds are more sensitive towards SUV alterations caused by technical variations as well as the application of new generation PET systems [39, 40]. Furthermore, measurements can be distorted due to the partial volume effect, particularly when using fixed thresholds [39, 41]. Apart from this, the limited sample size in our work and the study by Wang et al. might contribute to this finding.

Also, Wang et al. classified patients with stable disease on follow-up CT as non-responders [23]. However, reliable radiologic differentiation between post therapeutic fibrosis and remaining viable tumor cells is not possible [42]. Thus, in our study patients with stable tumor size in the follow-up CT were included in the responder cohort.

SUV_max_ is frequently determined in response assessment in clinical routine. In our study, it did not appear to be a single significant parameter with regard to chemotherapy response. This is in line with previous studies reporting SUV_max_ to be less reliable than volume-based PET-derived parameters with regard to the tumor burden [43–45]. SUV_max_ is strongly influenced by noise and the applied reconstruction algorithm [41]. Furthermore, it is derived from one voxel and thus not representing the entire tumor burden, which might explain our findings [46].

Previous studies on the performance of [^18^F]FDG PET/CT in PDAC revealed the potential of implementing metabolic imaging as a means to predict therapeutic response [27, 47, 48]. However, [^18^F]FDG PET/CT often suffers from poor tumor delineation on CT in comparison to [^18^F]FDG PET/MRI [22, 49, 50]. In fact, non-specific [^18^F]FDG uptake of the spleen and the duodenum might impede tumor delineation in [^18^F]FDG PET/CT [51, 52]. These shortcomings contribute to the known restraints of [^18^F]FDG in PDAC characterization, in particularly in the diagnostic work-up of patients with small tumors [53]. In this regard, fully-integrated [^18^F]FDG PET/MRI enables improved image fusion, reduction of motion artifacts and superior anatomic delineation [49, 54].

Moreover, PET/MRI enables the acquisition of functional MRI parameters such as the diffusion weighted imaging(DWI)-derived apparent diffusion coefficient (ADC). DWI plays an increasing role in pancreatic imaging, particularly with regard to PDAC [55]. Preclinical studies revealed DWI-derived ADC as a non-invasive biomarker for tumor cellularity which beyond that enables early response prediction in chemotherapy and radiation therapy [22, 26, 56, 57]. In our study, ADC_mean_ was a highly sensitive and specific single parameter for assessment of therapy response. In a multiparameteric analysis ADC_max_ enabled high to perfect discrimination in combination with TLG_50%_ and MTV_50%_, respectively.

Previous studies on [^18^F]FDG in PDAC revealed the potential of predicting survival and progression based on MTV and TLG [44, 58]. Because of the mixed nature of the cohort presented here, imaging parameters were not correlated with progression free or overall survival.

Our study has limitations. First, we performed a single institution study including only a small number of patients. In our study, both PET parameters and multiparametric combination of these with the MRI parameter yielded a ROC of 1, resulting in a perfect discrimination between responders and non-responders; however, this is likely due to the small cohort size. For future prospective validation of these findings, in consideration of limited availability of PET-MR units, [^18^F]FDG or diffusion weighted MRI alone may proof sufficient for response prediction with the benefits of simplified logistics and patient burden.

Second, we used RECIST1.1. to stratify our patient cohort in responders and non-responders. As previously reported RECIST is subject to various limitations [59]. Future prospective study design should aim for a broader inclusion of clinical meta-parameters (molecular tumor data, patient performance and co-morbidities).

Third, we observed a lower response rate in patients with a more advanced disease stage at baseline. This finding again, warrants further investigation in a larger follow up cohort, ideally including a best supportive care subgroup of patients and potentially an additional time point at regular follow-up (i.e. 3 months).

## Conclusion

In conclusion, our exploratory prospective study emphasizes the potential value of [^18^F]FDG PET/MRI -derived imaging parameters for early assessment of chemotherapy response in PDAC. We propose a large prospective multi-centric evaluation of the identified imaging parameters for improved patient stratification.

## Supplementary Information

**Additional file 1**. The STrengthening the Reporting of OBservational studies in Epidemiology (STROBE) flowchart is included in the supplementary material (A. 1).

## Abbreviations

ADC: Apparent diffusion coefficient
CA 19-9: Carbohydrate antigen 19-9
CEA: Carcinoembryonic antigen
CT: Computed tomography
DWI: Diffusion-weighted imaging
FDG: Fluorodeoxyglucose
FS: Fat saturated
LAPC: Locally advanced pancreatic cancer
MBq: Megabequerel
mPDAC: Metastatic pancreatic ductal adenocarcinoma
MR: Magnetic resonance
MRI: Magnetic resonance imaging
MTV: Metabolic tumor volume
PDAC: Pancreatic ductal adenocarcinoma
PET: Positron emission tomography
RECIST: Response evaluation criteria in solid tumors
ROC: Receiver operating characteristic
SUV: Standardized uptake value
TLG: Total lesion glycolysis
