# Supplementary material for: [18F]FDG PET/MRI enables early chemotherapy response prediction in pancreatic ductal adenocarcinoma
Source: EJNMMI Res. 2021 Jul 28;11:70. doi: 10.1186/s13550-021-00808-4 (PMC8319249; doi:10.1186/s13550-021-00808-4)
Supplement: Supplementary file 1 — Additional file 1. The STrengthening the Reporting of OBservational studies in Epidemiology (STROBE) flowchart is included in the supplementary material (A. 1). [file 13550_2021_808_MOESM1_ESM.docx]

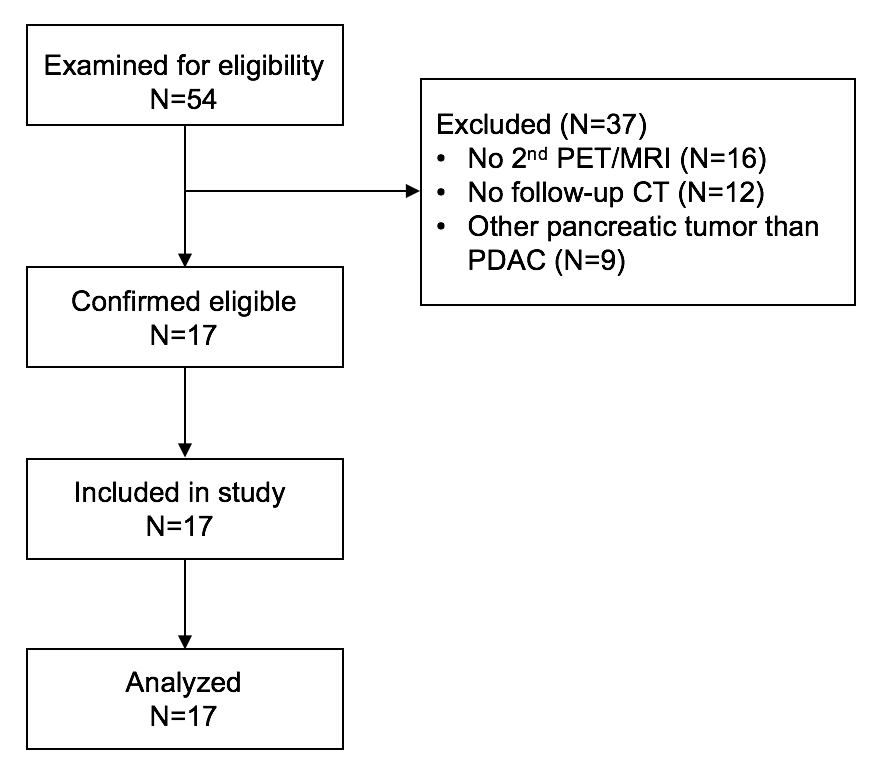


**A. 1** Patient inclusion flowchart.

| **Parameter** | **ax T2w HASTE** | **cor T2w HASTE** | **ax T2w HASTE FS** | **ax EPI DWI** | **ax T1w Vibe**  **pre CA** | **ax T1w Vibe Dynamic** | **ax T1w Vibe Venous** | **ax T1w Vibe Late Venous** | **ax T1w Vibe after CA** |
| --- | --- | --- | --- | --- | --- | --- | --- | --- | --- |
| **TR/TE (ms)** | 1600/95 | 1400/87 | 1600/95 | 5600/52 | 4.5/1.29/2.52 | 3.89/1.23/2.46 | 4.5/1.29/2.52 | 4.5/1.29/2.52 | 3.29/1.14 |
| **Flip angle (°)** | 160 | 160 | 160 | - | 9 | 10 | 9 | 9 | 9 |
| **FOV (mm)** | 380 | 400 | 300 | 380 | 380 | 400 | 380 | 380 | 460 |
| **Phase FOV (%)** | 75 | 100 | 78.1 | 80.6 | 81.3 | 100 | 81.3 | 81.3 | 75 |
| **Acceleration factor** | 2 | 3 | 2 | 2 | 3 | 3 | 3 | 3 | 2 |
| **Acquisition time (min)** | 1:08 | 0:54 | 1:14 | 3:52 | 0:17 | 0:17 | 0:17 | 0:17 | 0:13 |
| **Slice thickness (mm)** | 5 | 5 | 5 | 5 | 3 | 3 | 3 | 3 | 5 |
| **Fat saturation** | None | None | Yes | Yes | None | None | None | None | Yes |
| **Intravenous contrast** | None | None | None | None | None | Yes | Yes | Yes | Yes |
| **B-values (s/mm^2^)** | - | - | - | 0/50/300/600 | - | - | - | - | - |

**A. 2 MRI sequence parameters**

ax: axial; HASTE: *H*alf-Fourier *A*cquisition *S*ingle-shot *T*urbo spin *E*cho imaging; cor: coronal; FS: fat saturated; EPI: echo-planar imaging; DWI: diffusion-weighted imaging; VIBE: Volumetric interpolated breath-hold examination; CA: contrast agent; TR: repetition time; TE: echo time; ms: milliseconds; FOV: field-of-view

|  | **Responder**  **(mean ± SD)** | **Non-responder**  **(mean ± SD)** | **p-value** |
| --- | --- | --- | --- |
| **SUVmax (1)** | 0.24 ± 0.27 | 0.29 ± 0.22 | 0.5621 |
| **SUV peak (1)** | 0.28 ± 0.27 | 0.26 ± 0.19 | 0.8744 |
| **MTV50% (1)** | 0.29 ± 0.31 | 0.47 ± 0.36 | 0.9580 |
| **TGL 50% (1)** | 0.25 ± 0.21 | 0.34 ± 0.40 | 0.9580 |
| **MTV 2,5 (1)** | 0.40 ± 0.27 | 0.44 ± 0.37 | 0.9580 |
| **TLG 2,5 (1)** | 0.26 ± 0.2 | 0.36 ± 0.38 | 0.7921 |
| **SUVmax (2)** | 0.31 ± 0.28 | 0.30 ± 0.28 | 0.7921 |
| **SUV peak (2)** | 0.30 ± 0.26 | 0.31 ± 0.32 | 0.7921 |
| **MTV50% (2)** | 0.20 ± 0.17 | 0.65 ± 0.34 | **0.0177** |
| **TLG 50% (2)** | 0.13 ± 0.12 | 0.40 ± 0.36 | 0.0820 |
| **MTV 2,5 (2)** | 0.23 ± 0.22 | 0.41 ± 0.36 | 0.1876 |
| **TLG 2,5 (2)** | 0.17 ± 0.19 | 0.31 ± 0.39 | 0.4292 |
| **ACD mean (1)** | 0.48 ± 0.28 | 0.58 ± 0.28 | 0.6353 |
| **ADC min (1)** | 0.52 ± 0.30 | 0.76 ± 0.26 | 0.1023 |
| **ADC max (1)** | 0.34 ± 0.26 | 0.26 ± 0.21 | 0.4932 |
| **ADC mean (2)** | 0.51 ± 0.31 | 0.24 ± 0.20 | 0.1264 |
| **ADC min (2)** | 0.65 ± 0.22 | 0.38 ± 0.26 | 0.1264 |
| **ADC max (2)** | 0.58 ± 0.31 | 0.52 ± 0.22 | 0.7122 |
| **Age** | 0.79 ± 0.22 | 0.38 ± 0.25 | **0.0227** |
| **CEA** | 0.09 ± 0.29 | 0.09 ± 0.11 | 0.0782 |
| **CA19-9** | 0.13 ± 0.28 | 0.48 ± 0.34 | 0.1547 |

**A. 3** displays PET/MRI parameters from the first (1) and second (2) PET/MRI examination. Assessment of possible confounding PET/MRI parameters as well as clinical features (Age, CEA and CA 19-9) was performed using the Mann-Whitney U test.

|  | **Variable** | **Responder**  (n = 12) | **Non-responder**  (n = 5) | **p-value** |
| --- | --- | --- | --- | --- |
| **Sex** | Male  Female | 9 (75%)  3 (25%) | 1 (20%)  4 (80%) | 0.10 |
| **Tumor size** | cT1  cT2  cT3  cT4 | 0 (0%)  3 (25%)  1 (8%)  8 (67%) | 0 (0%)  2 (40%)  0 (0%)  3 (60%) | 0.60 |
| **Lymph node status** | cN0  cN1  cN2 | 5 (42%)  3 (25%)  4 (33%) | 1 (20%)  2 (40%)  2 (40%) | 0.60 |
| **Metastasis** | cM0  cM1 | 6 (50%)  6 (50%) | 1 (20%)  4 (80%) | 0.34 |
| **Type of chemotherapy** | FOLFIRINOX  gemcitabine based | 7 (58%)  5 (42%) | 4 (80%)  1 (20%) | 0.60 |

**A. 4** Assessment of possible confounding binary features using the fisher’s exact test.
